# Second Attack of Constitutional Syphilis

**Published:** 1880-09

**Authors:** 


### SECOND ATTACK OF CONSTITUTIONAL SYPHILIS.

Two cases reported at a meeting of the Medico-Chirurgical Society, of Louisville, March 5, 1880, by L. P. Yandell, M. D., and James M. Holloway, M. D.:

Dr. Yandell: A gentleman came to me two years ago in a cachectic condition, suffering from numerous and severe manifestations of secondary syphilis. He was a man of strumous diathesis, and a victim of malarial poisoning. Under quinine, iron, cod-liver oil, and malt, together with the moist mercurial vapor baths, he was, within a few months, entirely cured. His perfect recovery, when the condition described is considered (and I state further that he was beyond fifty years old), is rather remarkable. During the two following years he had no relapse—no syphilitic manifestation, though he often required iron, quinine, and sometimes the constructives. He got a prolonged course of iodide of potash after the baths.

A few months since he again consulted me for what his family physician considered *herpes*, but on pushing back the prepuce a semi-lunar, gristly induration, immediately behind the top of the glans penis, demonstrated that he was the victim of an indurated chancre. Under the same treatment that was used in the first instance, he is rapidly recovering. In something more than twenty years' practice, this is the first instance in which I have seen a second indurated chancre in the same individual; the first instance, at least, in which there is no possibility of mistake. If my memory serves me right, Mr. Jonathan Hutchinson, of London, told me he had seen several cases, and I have it in black and white from Ricord, in a note written to me some years since, that he has seen several cases of the kind.

Dr. Holloway: Three years ago a colored man, aged fifty-eight years, attended the surgical clinic at the Hospital College, and was treated during the entire year for consecutive syphilis. The students had an opportunity to observe in his case almost all of the consecutive manifestations of the disease upon the mucous and cutaneous membranes, and not a few upon the bones and nervous system and lymphatic system. Under a persistent mercurial treatment (internally), followed by iodide of potash, the patient, though advanced in years, slowly recovered. While he was under treatment his wife was also a dispensary patient with the same disease, as was also his son, aged about eighteen years. The notes of my lectures during this time, taken by the advanced students, abound in references to these cases, especially that of the old man. He was otherwise healthy—free from the strumous and malarial complications referred to by Dr. Yandell. After his recovery he visited the dispensary frequently upon his own account, but oftener to introduce other patients. On these occasions his case was cited as a probable recovery from constitutional syphilis.

Last winter this man presented himself again for inspection, and upon a careful and thorough examination was proved to be the possessor of an indurated, split-pea-like chancre, which was located upon the preputial mucous membrane, near the corona glandis. The mucous membrane of the glans and prepuce and a narrow circle of the contiguous skin were free from pigment, rendering the appearance of the chancre identical with those of a white man. The inguinal lymphatics were symmetrically enlarged and painless.

This patient was given a mercurial course (calomel and opium internally), and directed to keep the sore dry and clean. After three weeks the chancre had disappeared. There had been no secondary symptoms three months after.

These cases prove either that one attack of constitutional syphilis does not give immunity from a second attack, or that the disease is curable. I entertain the latter opinion.

Such cases should be brought prominently before the profession. Frequently well-informed practitioners express doubts as to the curability of syphilis, and much oftener do I hear similar doubts expressed by non-professional persons. I not only believe firmly in the curability of syphilis by appropriate and judicious medication, but I have almost conclusive evidence that the disease is occasionally recovered from without any treatment whatever further than that denominated hygienic.—*Louisville Medical News*, May 22.

